# Leveraging electronic health records to identify risk factors for recurrent pregnancy loss across two medical centers: a case-control study

**DOI:** 10.21203/rs.3.rs-2631220/v2

**Published:** 2023-03-31

**Authors:** Jacquelyn Roger, Feng Xie, Jean Costello, Alice Tang, Jay Liu, Tomiko Oskotsky, Sarah Woldemariam, Idit Kosti, Brian Le, Michael P. Snyder, Linda C. Giudice, Dara Torgerson, Gary M. Shaw, David K. Stevenson, Aleksandar Rajkovic, M. Maria Glymour, Nima Aghaeepour, Hakan Cakmak, Ruth B. Lathi, Marina Sirota

**Affiliations:** 1. Bakar Computational Health Sciences Institute, University of California San Francisco; 2. Department of Anesthesiology, Perioperative, and Pain Medicine, Stanford University; 3. Department of Pediatrics, Stanford University; 4. Department of Biomedical Data Science, Stanford University; 5. Department of Genetics, Stanford University School of Medicine; 6. Department of Obstetrics and Gynecology, University of California San Francisco; 7. Department of Epidemiology and Biostatistics, University of California San Francisco; 8. Department of Pathology, University of California San Francisco; 9. Institute of Human Genetics, University of California San Francisco; 10. Department of Obstetrics and Gynecology, Stanford University

**Keywords:** recurrent pregnancy loss, EMR, clinical phenotyping

## Abstract

Recurrent pregnancy loss (RPL), defined as 2 or more pregnancy losses, affects 5–6% of ever-pregnant individuals. Approximately half of these cases have no identifiable explanation. To generate hypotheses about RPL etiologies, we implemented a case-control study comparing the history of over 1,600 diagnoses between RPL and live-birth patients, leveraging the University of California San Francisco (UCSF) and Stanford University electronic health record databases. In total, our study included 8,496 RPL (UCSF: 3,840, Stanford: 4,656) and 53,278 Control (UCSF: 17,259, Stanford: 36,019) patients. Menstrual abnormalities and infertility-associated diagnoses were significantly positively associated with RPL in both medical centers. Age-stratified analysis revealed that the majority of RPL-associated diagnoses had higher odds ratios for patients <35 compared with 35+ patients. While Stanford results were sensitive to control for healthcare utilization, UCSF results were stable across analyses with and without utilization. Intersecting significant results between medical centers was an effective filter to identify associations that are robust across center-specific utilization patterns.

## Introduction

Pregnancy loss is the most common pregnancy complication, occurring in almost a third of all clinically recognizable pregnancies^1–3^. Pregnancy loss is an umbrella term that encompasses any pregnancy that does not progress to live-birth and includes both miscarriage and stillbirth, if the demise occurs before or after 20 weeks gestation respectively. Recurrent pregnancy loss (RPL), defined as two or more losses, is also common; an estimated 5–6% of ever-pregnant individuals and up to 16% of parous individuals experience RPL^4,5^. RPL can be devastating for pregnant individuals and their families^6–8^, and is associated with a poorer prognosis for subsequent pregnancies^9^.

RPL is a complex and multifactorial condition. Known causes include maternal age over 35 years, numerical or segmental chromosomal abnormalities, congenital uterine abnormalities, antiphospholipid antibody syndrome, and uncontrolled hormonal or metabolic conditions^10^. Several studies have uncovered additional associations with RPL. These studies have been largely hypothesis-driven, as the association testing was restricted to a handful (or fewer) of potential risk factors. From these studies, a range of RPL associations have been reported: lifestyle factors^11–13^, genetic variation^14^, infertility-associated diagnoses^15–17^, hereditary thrombophilias^18–20^, infections^21–23^, and environmental exposures^24–28^. There is also increasing evidence of the role of paternal health in pregnancy loss^29^, including sperm DNA fragmentation^30,31^. Still, approximately half of all RPL patients have no identifiable causes for their losses^32–34^. This substantial gap suggests that our current understanding of RPL etiologies is incomplete.

Electronic health record (EHR) databases contain multimodal longitudinal data on patients. Computational methods provide the opportunity to mine this data and uncover patterns within patient populations. Previous projects analyzing EHR data to study RPL have investigated disease incidence after RPL^35^ and characterized lifetime phenotypic associations of idiopathic RPL^14^. The first study included 10,691 RPL patients from Denmark, and reported an increased risk of cardiovascular and gastrointestinal disorders later in life, for primary and secondary RPL patients respectively^35^. The second study included 458 idiopathic RPL patients from the United Kingdom Biobank (UKBB), and reported that patients with idiopathic RPL were more likely to be diagnosed with tubulointerstitial nephritis, infertility, or ectopic pregnancy anytime in their lifetimes^14^.

To our knowledge, there have not been any EHR studies focused on identifying potential risk factors for RPL. Such a study could generate novel hypotheses about RPL etiologies, reveal new avenues for RPL prevention research, and guide future predictive modeling to identify patients at high risk for RPL.

**Figure 1:**
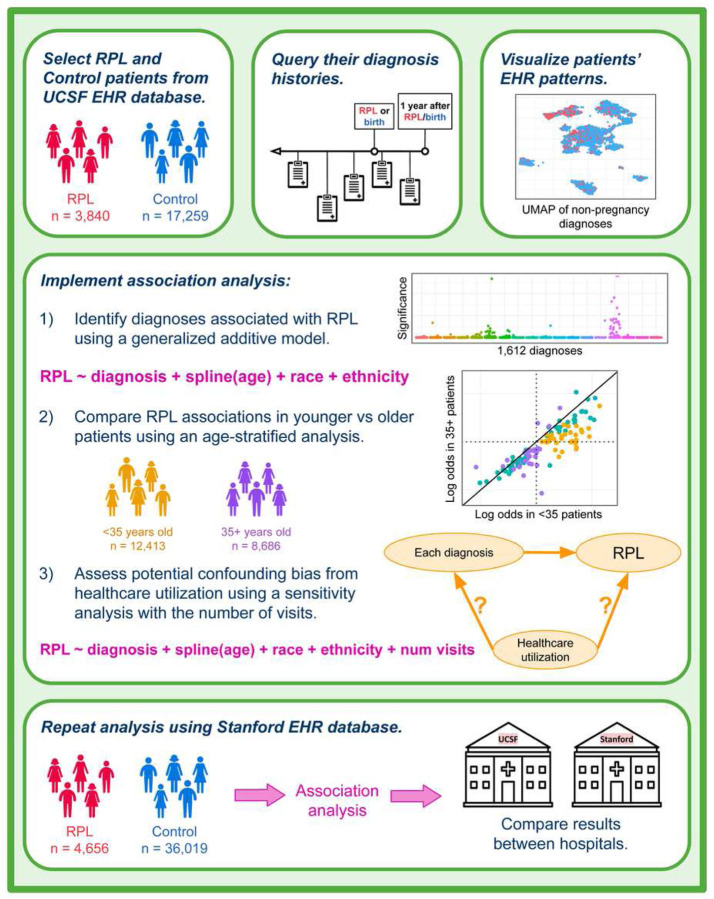
Study design. Patients were selected from the UCSF OMOP EHR database by querying for records of RPL (for RPL group) or live-birth (for Control group). For all patients selected, we queried their diagnosis histories prior to their first record of RPL/birth up until a year afterwards. We visualized patients’ HER patterns using a UMAP of their non-pregnancy-related diagnoses. Three analyses were implemented: (1) main association analysis, (2) age-stratified analysis, and (3) healthcare utilization sensitivity analysis. All queries and analyses were repeated using the Stanford EHR database for external validation.

To achieve this goal, we implemented a case-control study comparing the frequency of over 1,600 candidate diagnoses in RPL and live-birth patients, leveraging de-identified EHR data from the University of California San Francisco (UCSF) and Stanford University databases. All diagnoses occurring before RPL onset (or first live-birth for Control patients) up until a year following RPL onset (or birth) were considered. The year afterwards was included because sometimes patients undergo diagnostic evaluations following RPL (or birth) that are relevant for understanding their pregnancy outcomes. We also visualized patients’ EHR patterns and implemented several additional analyses (Figure 1). To compare RPL associations in younger vs older patients, we carried out an age-stratified analysis of patients under 35 years of age and patients 35 years and older. In addition, we hypothesized that RPL patients may have increased healthcare utilization relative to Control patients, which could lead to increased recognition of pregnancies and incidental diagnoses. We therefore conducted a sensitivity analysis controlling for the number of visits as an indicator of utilization. Lastly, to ascertain which results validated across medical centers, we compared findings from our UCSF and Stanford studies. This validation work builds on our existing foundation of porting EHR analysis models between medical centers towards generalizability of both methods and results^36^.

Here, we analyzed the diagnostic histories of 8,496 RPL patients, 53,278 Control patients, and over 1,600 diagnoses from two independent medical centers to identify diagnoses positively and negatively associated with RPL. To our knowledge, this is the first RPL EHR study focused on risk factors occurring before or near RPL onset, with the goal of generating hypotheses about RPL etiologies.

## Results

### Description of RPL and Control patients

**Figure 2:**
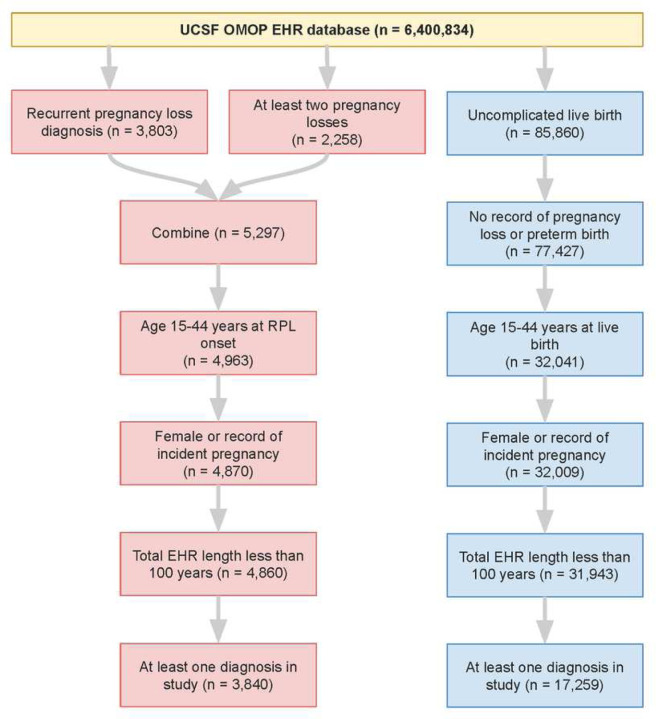
Patient selection at UCSF. In total, 3,840 RPL patients were selected (red) and 17,259 Control patients were selected (blue).

We identified RPL and Control patients in the UCSF and Stanford de-identified EHR databases by querying for concepts indicating pregnancy losses or live-birth (see Methods, Supplementary File 1). From the UCSF EHR database containing 6.4 million patients, 3,840 RPL and 17,259 Control patients were selected (Figure 2). From the Stanford EHR database containing 3.6 million patients, 4,656 RPL and 36,019 Control patients were selected (Supplementary Figure 1).

**Figure 3:**
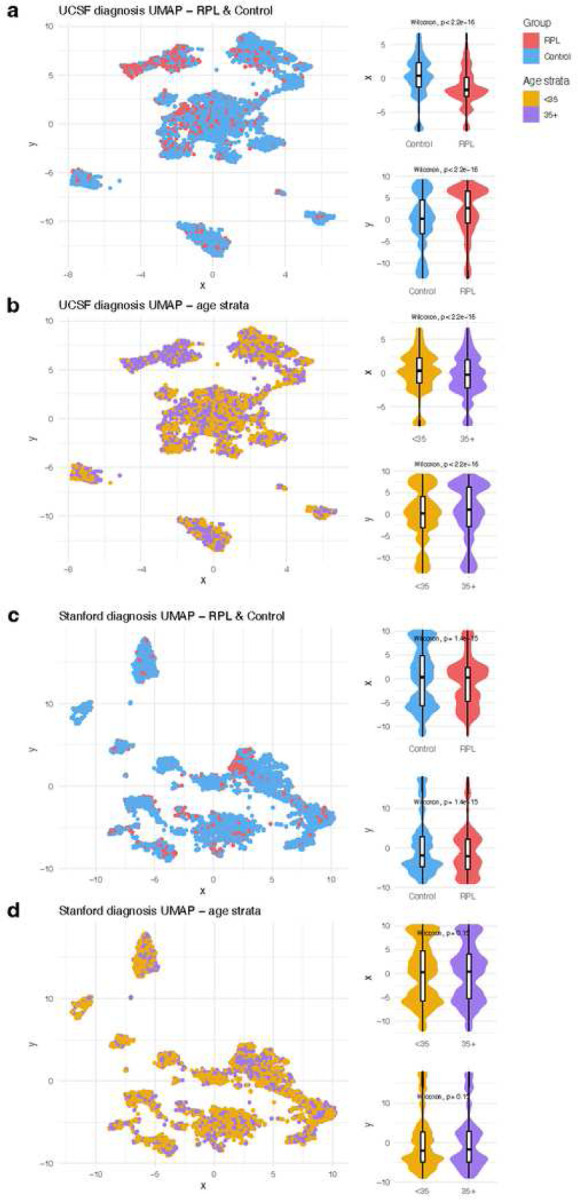
UMAP of non-pregnancy diagnoses. We applied UMAP (a dimensionality reduction algorithm) to visualize each patient’s diagnosis patterns. Diagnoses in the “pregnancy complications” category were excluded from the UMAP to ensure that the subsequent dimensionality reduction was based on diagnoses leading to the outcome (RPL or live-birth), instead of diagnoses indicating the outcome. Age strata (<35 vs 35+) were also compared.

UCSF patients’ demographics and healthcare utilization distributions are summarized in Table 1. Overall, RPL patients were older than Control patients (median age: 36.6 vs 33.4, t-test *p*-value<0.001). Fewer RPL patients were identified as Hispanic or Latino compared with Control patients (11.7% vs 16.6%, chi-square test *p*-value<0.001). RPL patients had slightly more visits (median 42.5 vs 41, t-test *p*-value<0.001), longer EHR records (median 3.44 vs 2.04 years, t-test *p*-value<0.001), and fewer diagnoses (median 9 vs 13, t-test *p*-value<0.001). To visualize patients’ EHR patterns, we applied Uniform Manifold Approximation and Projection (UMAP) to their non-pregnancy diagnoses and then plotted the patients’ coordinates in two-dimensional space. Plots were colored by outcome (Figure 3a) and age strata (Figure 3b). Coordinate distributions were significantly different across patient outcomes and age strata. UCSF UMAP plots colored by race, ethnicity, number of visits, years in EHR, and number of diagnoses are included in Supplementary Figures 2–3.

Stanford patients’ demographics and healthcare utilization distributions are summarized in Supplementary Table 1. Overall, RPL patients were older than Control patients (median age 35.4 vs 32.4, t-test *p*-value<0.001). Fewer RPL patients were identified as Hispanic or Latino compared to Control patients (16.4% vs 29.3%, chi-square test *p*-value<0.001). RPL patients had more visits (median 31 vs 14, t-test *p*-value<0.001), longer EHR records (median 3.14 vs 1.67 years, t-test *p*-value<0.001), and more diagnoses (median 11 vs 9, t-test *p*-value<0.001). We visualized the EHR patterns of Stanford patients across outcomes (Figure 3c) and age strata (Figure 3d). Coordinate distributions were significantly different across patient outcomes, but not significantly different across age strata. Stanford UMAP plots colored by race, ethnicity, number of visits, years in EHR, and number of diagnoses are included in Supplementary Figures 4–5.

To enable more accurate gender reporting, patients’ gender identities were derived from a combination of gender and diagnostic queries (see Methods). At UCSF, 99.9% of patients were cisgender women and 0.1% were transgender, non-binary, or gender-diverse. At Stanford, >99.9% of patients were cisgender women and <0.1% were transgender, non-binary, or gender-diverse. At both UCSF and Stanford, the gender distributions were not significantly different between RPL and Control groups (chi-square test *p*-value>0.05).

### Menstrual abnormalities and infertility-associated diagnoses were positively associated with RPL

For the association analysis, we evaluated whether each candidate diagnosis was positively or negatively associated with RPL. Positive associations suggest potential risk factors for RPL. Negative associations either suggest potential protective factors for RPL or reflect diagnoses that occur more frequently in live-birth (Control) patients.

Any diagnosis occurring at least once in an RPL or Control patient’s EHR during the study window was included as a candidate diagnosis. ICD diagnostic codes were mapped to Phecodes (see Methods) resulting in 1,612 and 1,662 candidate diagnoses at UCSF and Stanford, respectively. Each candidate diagnosis was tested for its association with RPL using a generalized additive model (GAM), with maternal age, race, and ethnicity included as covariates. In each association analysis, all *p*-values were adjusted for multiple testing using the Benjamini-Hochberg method to reduce the false discovery rate.

**Figure 4:**
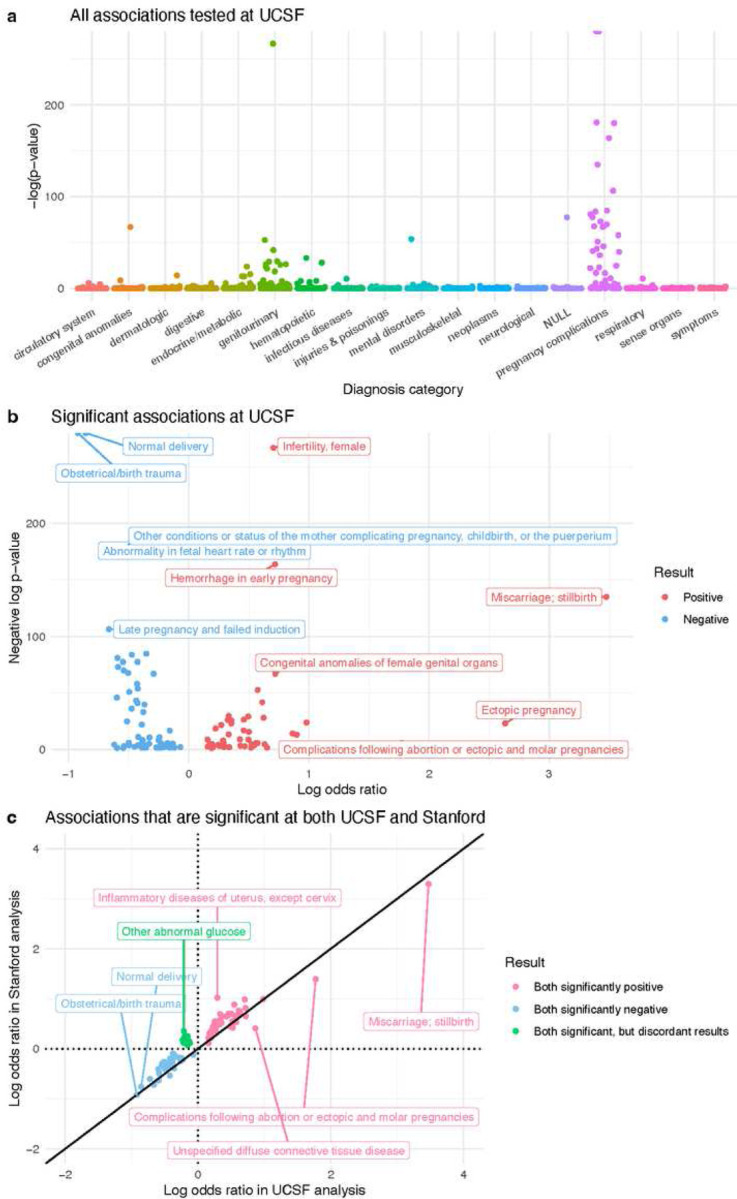
UCSF association analysis results. (a) Manhattan plot of all diagnoses tested in the UCSF analysis. Diagnosis categories are listed on the x-axis. The y-axis is the negative log of each associations’ p-value, from each diagnosis’s GAM. (b) Volcano plot of significant (p-value<0.05) associations. In the Manhattan and Volcano plots, a few diagnoses have negative log p-values that are approaching infinity, so their corresponding points are located on the top border of the plot. (c) Log-Log plot of validated positive, validated negative, and discordant association results. These diagnoses are significant in both the UCSF and Stanford analyses. All points in the Log-Log plots were filtered to include diagnoses where ≥10 patients in either the RPL or Control group have a record of that diagnosis. All log transformations in these plots are in base 10.

At UCSF, 1,612 candidate diagnoses were tested (Figure 4a, Supplementary File 2). 120 were found to have a significant relationship (*p*-value<0.05) with RPL: 51 diagnoses were positively associated with RPL and 69 were negatively associated with RPL (Figure 4b). At Stanford, 1,662 diagnoses were tested (Supplementary Figure 6a, Supplementary File 3). 367 showed a significant relationship with RPL: 330 positive and 37 negative (Supplementary Figure 6b). Overall, Stanford had a higher proportion of positive associations than UCSF (330/367 vs 51/120, respectively).

In total, 1,576 candidate diagnoses were shared across the UCSF and Stanford analyses. To determine which results validated across medical centers, we computed the intersection of significant results from the UCSF and Stanford analyses. We found that 88 diagnoses were significantly associated with RPL at both sites, and their odds ratios were very highly correlated between sites (Spearman r=0.946, *p*-value<0.001). Of those 88 diagnoses, 42 diagnoses were positive in both, 34 were negative in both, and 12 were discordant (significant in both, but in opposite directions). We applied a hypergeometric test to ascertain whether the overlap of significantly positive diagnoses (42/1,576) between UCSF and Stanford was greater than what we would expect by chance. This test was repeated for the overlap of significantly negative diagnoses (34/1,576) as well. Both overlaps were significant (both *p*-values<0.001 from their respective hypergeometric tests).

Inter-center validated positive association results are summarized in Table 2. At both UCSF and Stanford, we replicated known RPL associations including chromosomal anomalies, congenital anomalies of genital organs, primary hypercoagulable state, and hemorrhage in early pregnancy. Our association analysis also uncovered several menstrual abnormalities: absent/infrequent menstruation, excessive/frequent menstruation, irregular menstrual cycle/bleeding, and other menstrual disorders. Odds ratios for menstrual abnormalities ranged from 1.67–2.67 in the UCSF analysis and 2.74–4.95 in the Stanford analysis (all *p*-values<0.001 from their respective GAMs).

Several infertility-associated diagnoses were positively associated with RPL. This includes both the broad “infertility” diagnosis and more specific infertility-associated diagnoses such as endometriosis, polycystic ovaries (PCOS), ovarian dysfunction, and ovarian failure. Odds ratios for infertility-associated diagnoses ranged from 2.15–5.07 in the UCSF analysis and 2.73–9.69 in the Stanford analysis (all *p*-values<0.001 from their respective GAMs).

Positive association results also include infections, immunological conditions, ovarian cyst, and dysmetabolic syndrome X (more commonly called “metabolic syndrome”). Inter-center validated negative association results primarily consist of diagnoses related to childbirth and pregnancy (Table 3). Discordant association results include diagnoses related to mental health, glucose, and pregnancy (Table 4).

### RPL-associated diagnoses had higher odds ratios for patients <35 compared with patients 35+

Maternal age plays a key role in pregnancy loss risk. The risk starts increasing slightly around age 30, and increases dramatically around age 35^37^. Age is also an important factor for virtually every aspect of health. To compare RPL association patterns in younger vs older patients, we implemented an age-stratified analysis with two age strata: <35 and 35+ years. Demographics and healthcare utilization for <35 and 35+ patients at UCSF and Stanford are reported in Supplementary Tables 2–5.

**Figure 5:**
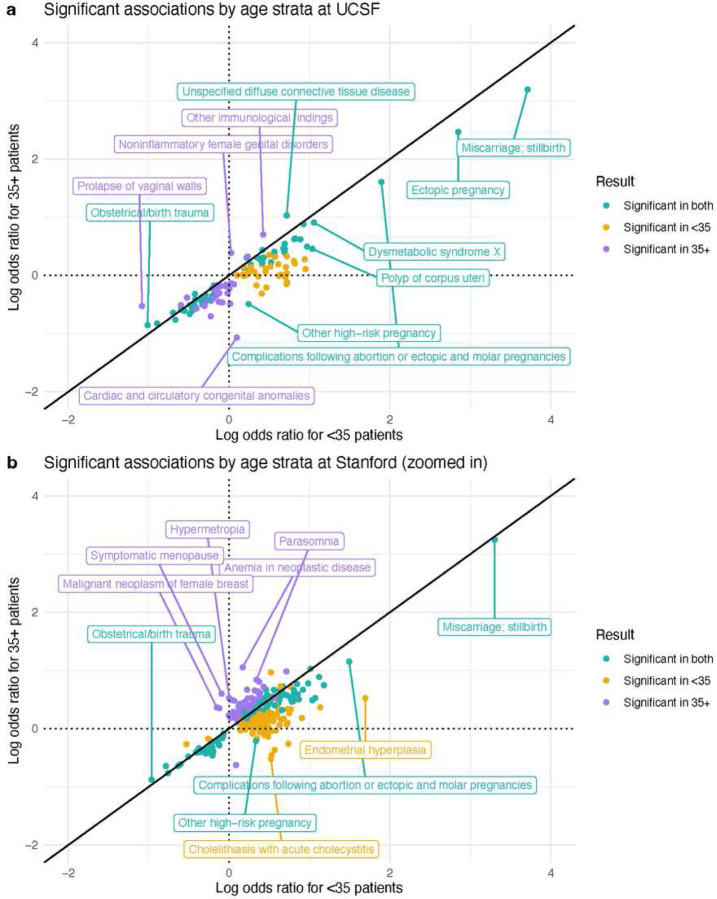
Age-stratified analysis results. (a) At UCSF. (b) At Stanford (zoomed in for readability). The full Stanford plot with outliers is in Supplementary Figure 7. All points in the Log-Log plots were filtered to include diagnoses where ≥10 patients in either the RPL or Control group have a record of that diagnosis. All log transformations in these plots are in base 10.

In the UCSF age-stratified analysis, the associations of 1,419 candidate diagnoses with RPL were evaluated in both the <35 and 35+ strata (Supplementary File 2). To compare results between age strata, we computed the union of significant results from both the <35 and 35+ analyses. We found that 128 diagnoses were significant in at least one age stratum. While their odds ratios were ordinally correlated between strata (Spearman r=0.889, *p*-value<0.001), the vast majority (111/128) of odds ratios were higher in <35 patients compared with 35+ patients. Clinically intriguing examples include polyp of corpus uteri, metabolic syndrome, high-risk pregnancy, and complications following abortion or ectopic & molar pregnancies (Figure 5a).

In the Stanford age-stratified analysis, 1,512 diagnoses were tested in both the <35 and 35+ strata (Supplementary File 3). Of those, 342 were significant in at least one stratum. Their odds ratios were modestly correlated (Spearman r=0.365, *p*-value<0.001), and the majority (239/342) of odds ratios were higher in <35 patients compared with 35+ patients. Clinically intriguing examples include high-risk pregnancy and complications following abortion or ectopic & molar pregnancies (Figure 5b).

### While Stanford results were sensitive to control for healthcare utilization, UCSF results were stable across analyses

In our healthcare utilization sensitivity analysis, we assessed whether controlling for healthcare utilization changed our association results. To do this, we re-estimated associations, adding a covariate for patients’ number of visits during the study window (anytime before RPL/birth up until a year after RPL/birth). The number of visits was selected as a metric for healthcare utilization because it is a direct measure of how much contact a patient has had with the healthcare system. Then, we calculated the median percent difference between odds ratios without and with the number of visits included in the model.

**Figure 6:**
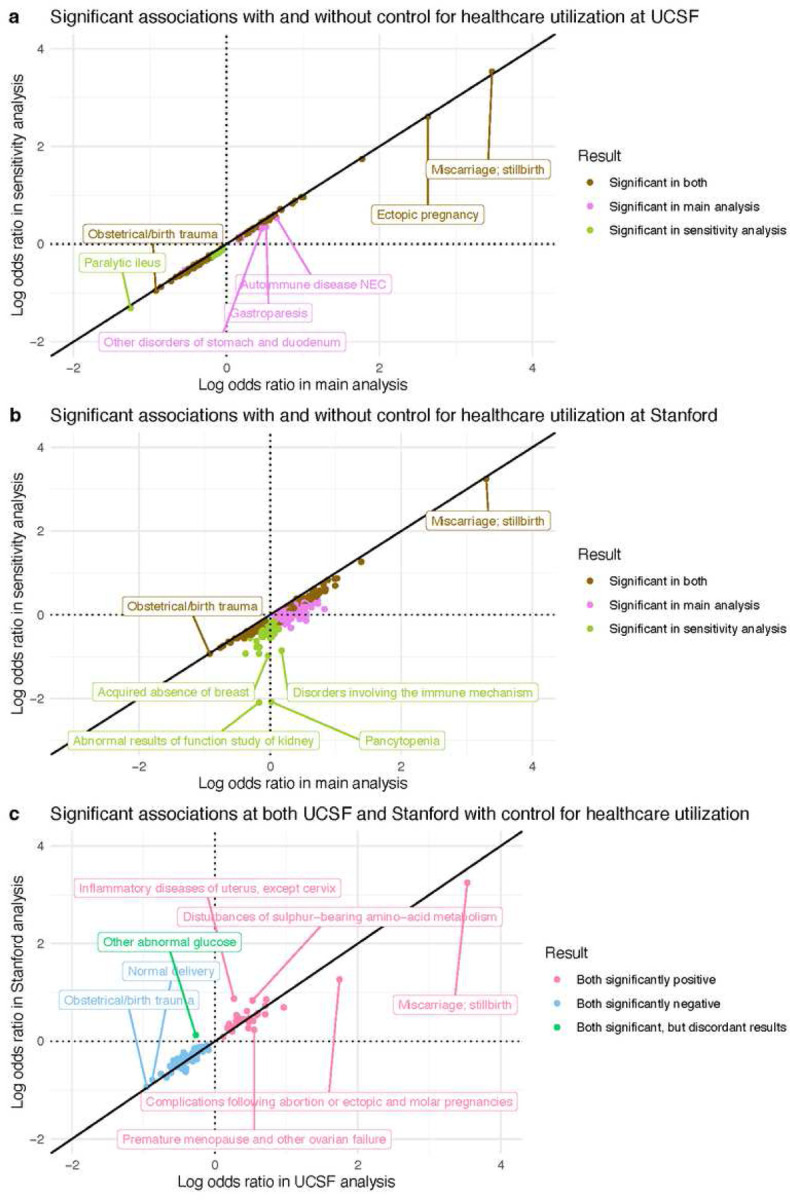
Healthcare utilization sensitivity analysis results. (a) At UCSF. (b) At Stanford. (c) Validated positive, validated negative, and discordant association results, after accounting for healthcare utilization in both the UCSF and Stanford analyses. All points in the Log-Log plots were filtered to include diagnoses where ≥10 patients in either the RPL or Control group have a record of that diagnosis. All log transformations in these plots are in base 10.

In the UCSF healthcare utilization sensitivity analysis, 138/1,612 diagnoses were significant: 42 positive and 96 negative. To compare association results between UCSF’s main analysis and UCSF’s sensitivity analysis, we computed the union of significant results from both. 148 diagnoses were significant in either analysis (Figure 6a). Their odds ratios were extremely highly correlated (Spearman r=0.997, *p*-value<0.001). After adjusting for healthcare utilization, odds ratios decreased modestly (median 13% decrease). Adjusted odds ratios and *p*-values from the UCSF sensitivity analysis are reported in Supplementary File 2.

In the Stanford healthcare utilization sensitivity analysis, 162/1,662 diagnoses were significant: 56 positive and 106 negative. Computing the union of significant results from Stanford’s main and sensitivity analysis yielded 421 diagnoses significant in either analysis (Figure 6b). Their odds ratios had very high ordinal correlation (Spearman r=0.912, *p*-value<0.001). However, the odds ratios’ values were quite different: after adjusting for healthcare utilization, odds ratios decreased substantially (median 49% decrease). Adjusted odds ratios and *p*-values from the Stanford sensitivity analysis are reported in Supplementary File 3.

In summary, healthcare utilization was associated with RPL, as evidenced by the reduction in significantly positive associations and their respective odds ratios, compared to the main models. These reductions were minor in the UCSF analysis and major in the Stanford analysis. However, significant results between the main and sensitivity analyses were highly correlated.

### Most of the inter-center validated results were sustained in models controlled for utilization

To ascertain which association results validated across medical centers after controlling for number of visits, we computed the intersection of significant results from both medical centers’ healthcare utilization sensitivity analyses (Figure 6c). 90 diagnoses were significant in both centers, and their odds ratios were very highly correlated (Spearman r=0.944, *p*-value<0.001). Of those 90 diagnoses, 33 diagnoses were positive in both, 56 were negative in both, and 1 was discordant (significant in both, but in opposite directions).

Next, we assessed whether the inter-center validated results from the main analysis were sustained in the models controlled for utilization. Of the 42 validated positive associations from the main analysis, 33 were also significantly positively associated with RPL at both UCSF and Stanford after controlling for utilization. All 34 validated negative associations from the main analysis were sustained in this sensitivity analysis.

## Discussion

We conducted a large-scale association analysis to identify diagnoses positively or negatively associated with RPL in two independent EHR databases. Additionally, we compared RPL associations in younger vs older patients using an age-stratified analysis, and assessed potential confounding from healthcare utilization using a sensitivity analysis with the number of visits. Our positive association results replicated several known RPL associations spanning chromosomal, anatomical, endocrine, and coagulation conditions (Table 2a). We also uncovered several potentially novel and clinically intriguing positive associations, described below.

A constellation of menstrual abnormalities were positively associated with RPL, ranging from absent/infrequent menstruation to excessive/frequent menstruation (Table 2b). Two smaller studies on menstrual abnormalities and pregnancy loss reported positive associations^38,39^. However, both of their association results were non-significant, possibly due to being under-powered. The first study (N=2,046) found a non-significant positive association between short (≤25 days) menstrual cycle length and pregnancy loss^38^. The second study (N=252) reported that individuals with short (<10 days) luteal phases in all three pre-conception menstrual cycles had a non-significant increased risk of pregnancy loss^39^. Our study included a much larger patient population (N=61,774), and all our reported menstrual abnormality results were quite significant (all *p*-values<0.001 from their respective GAMs).

Menstrual abnormalities could be important risk factors for RPL. They are easily screened and monitored over time, especially with the rise of menstrual health apps^40^. Additionally, there are several potential mechanisms for menstrual abnormalities to be associated with pregnancy loss. They could signal inadequate progesterone production^41^, which could be caused by thyroid or ovarian dysfunction^41–44^. There are also structural causes of menstrual abnormalities, including fibroids, polyps, or other uterine anomalies^45–47^. Depending on the clinical workup that RPL patients receive, some of them may have undiagnosed hormonal or structural abnormalities, but present as abnormal menses.

In our study, both infertility and infertility-associated diagnoses (e.g., endometriosis, PCOS, ovarian dysfunction/failure) were positively associated with RPL (Table 2c). While RPL is the inability of pregnancies to reach viability, infertility is the inability to conceive a pregnancy. There is longstanding debate about whether RPL and infertility are connected^48–51^. Additionally, a previous RPL EHR study of UKBB data also reported a positive association between RPL and infertility^14^. However, in EHR data, the association between the broad “infertility” diagnosis and RPL may be confounded by RPL patients incorrectly receiving an infertility diagnosis instead of an RPL diagnosis. For example, pregnancies may be lost before they are recognized, leading to inaccurate infertility diagnoses. Additionally, physicians who do not specialize in obstetrics may consider RPL a type of infertility, whereas OB-GYN physicians would classify these as two separate reproductive conditions. However, this misclassification is unlikely to occur for the more specific infertility-associated diagnoses (endometriosis, PCOS, ovarian dysfunction/failure).

Several prior studies have investigated potential associations between RPL and infertility-associated diagnoses. Our endometriosis findings are consistent with a recent nationwide cohort study in Denmark that found a significant positive association between endometriosis and pregnancy loss, including stronger associations with increasing number of losses^52^. Prior literature on a relationship between PCOS and RPL is mixed, with a couple of studies reporting significant positive associations^15,53^ and another reporting no significant association^54^. Our PCOS results provide further evidence of a positive association between PCOS and RPL. There is limited previous literature on an association between ovarian failure/dysfunction and pregnancy loss, likely due to low conception rates within those patients. A 1999 systematic review found that primary ovarian failure patients had a pregnancy loss rate comparable to the general population^55^. However, in our study, we observed a significant positive association with RPL for both ovarian dysfunction and ovarian failure. There are a couple potential mechanisms for this association. For patients with diminished ovarian reserves, their remaining eggs may be less likely to be euploid^56^, which could contribute to aneuploidy-related RPL^57^. Additionally, hormonal production dysregulation in patients with ovarian dysfunction/failure could contribute to pregnancy loss risk.

Our results for vaginitis/vulvovaginitis and pelvic inflammatory disease (Table 2d) suggest that the vaginal and uterine microbiome could play a role in pregnancy loss risk. While vaginal microbiome composition has been widely studied in the context of preterm birth^58–61^, its role in pregnancy loss is less understood. Two previous studies reported that vaginal microbiome composition was associated with both pregnancy loss^62^ and history of pregnancy loss^63^. History of pregnancy loss has also been associated with bacterial vaginosis and vulvovaginal candidiasis^64^. Lastly, there is evidence that untreated chronic endometritis can contribute to pregnancy loss risk^65–67^. In aggregate, our results and those of previous studies indicate that the vaginal and uterine microbiome may be important for unraveling RPL etiology.

Metabolic syndrome was very positively associated with RPL at both UCSF and Stanford (Table 2e). A relationship between these two conditions has been previously reported^68^, with one study hypothesizing that metabolic syndrome mediates inflammatory and oxidative stress responses in RPL^69^. However, another study did not find any significant associations between metabolic parameters and RPL^70^. Additionally, metabolic syndrome is very common in patients with PCOS^71^, which has also been associated with RPL. Further studies are needed to discern whether PCOS is confounding the association between metabolic syndrome and RPL, or whether there is a real link between metabolic syndrome and RPL.

The negative association results were mainly related to childbirth/pregnancy (Table 3). Most of these conditions occur in the second trimester, so they were presumably from pregnant individuals in the Control group. The replication of known associations with pregnancy and live-birth are further affirming of our overall approach.

There were a handful of diagnoses with discordant results. While their *p*-values were significant at both UCSF and Stanford, their odds ratios were in opposite directions – they were negatively associated with RPL at UCSF and positively associated with RPL at Stanford. Discordant results included anxiety, depression, impaired glucose, and abnormal glucose (Table 4). These discordances are most likely due to differences in screening practices between the two medical centers. For example, the relative frequency of routine mental health screenings for RPL vs live-birth patients may be different between UCSF and Stanford.

In our age-stratified analysis, odds ratios tended to be higher in <35 patients vs 35+ patients (Figure 5). Age plays an increasing role in pregnancy loss risk the older that patients get^37^, so this could result in other factors playing a decreasing role the older that patients get. This tendency was less pronounced at Stanford (Figure 5b) compared with UCSF (Figure 5a). Additionally, in the UMAP visualizations, there was less separation between age strata at Stanford (Figure 3d) than at UCSF (Figure 3b). Future studies could investigate the nature of age-related patterns for RPL-associated diagnoses, including testing for effect modifications or interactions.

Controlling for healthcare utilization as measured with visit count reduced both the proportion of significantly positive associations and the odds ratios for significant diagnoses. These reductions were small at UCSF (Figure 6a) but larger at Stanford (Figure 6b). This is likely because the difference in utilization levels between RPL vs Controls was much more pronounced at Stanford than at UCSF. Stanford RPL patients had many more visits than Stanford Control patients (median: 31 vs 14) (Supplementary Table 1). However, UCSF RPL patients had a similar number of visits as UCSF Control patients (median 42.5 vs 41) (Table 1).

We controlled for healthcare utilization in sensitivity analyses rather than primary results because utilization can be either a cause or a consequence of many of the candidate diagnoses we evaluated. Without greater temporal resolution, it is therefore unclear whether analyses should be adjusted for utilization. For example, patients with higher utilization may be more likely to receive an RPL diagnosis and any other diagnoses^72^. To address this confounding bias, we can adjust for utilization by adding it as a covariate in our models. However, including utilization in our models could *lead* to an underestimation of the association between each candidate diagnosis and RPL (i.e., collider bias), because having diagnoses such as menstrual abnormalities, infertility, or PCOS may increase healthcare utilization. Thus, it is useful to compare models with and without utilization to assess potential confounding bias, but utilization was not included in our main model to avoid introducing collider bias.

These findings demonstrate that healthcare utilization can play a minor role in some studies (e.g., UCSF analysis) and a major role in other studies (e.g., Stanford analysis), so it must always be evaluated carefully. The dramatic change in Stanford results after controlling for utilization illustrates how sensitive effect size estimates can be in EHR association analyses when the comparison groups have very different utilization levels. However, it is important to note that the changes in effect estimates should not be interpreted as exact measures of confounding. Rather, these changes reflect a combination of confounding and noncollapsibility^73^.

Overall, it was affirming to observe that most of the inter-center validated results were sustained in models controlled for utilization. This illustrates the utility of external validation for identifying associations that are robust across center-specific healthcare utilization patterns. Moreover, it was affirming that the association results were stable in the UCSF data when comparing between the main vs sensitivity analysis (Figure 6a). This suggests that healthcare utilization is not a major confounder or collider in the UCSF analyses.

A large and diverse patient population was included in our study (Table 1, Supplementary Table 1). We observed significant differences in the distributions of patients’ identified races and ethnicities between RPL and Control groups at both UCSF and Stanford. In particular, fewer RPL patients were identified as Hispanic or Latino compared with Control patients (UCSF: 11.7% vs 16.6%, Stanford: 16.4% vs 29.3%). Future studies could further examine these differences, with the goal of identifying interventions to reduce any disparities in pregnancy loss risk and clinical care^74,75^.

Our study has several strengths. This is the first RPL clinical association study to focus on diagnoses occurring before and near RPL onset, with the goal of generating hypotheses about RPL etiologies. We included a large patient population, with 8,496 RPL and 53,278 Control patients. Methodologically, this is the only RPL clinical association analysis whose findings were externally validated in a separate EHR database, and the only RPL clinical association analysis to assess the sensitivity of results to healthcare utilization. Lastly, we provide a framework including phenotype definitions (Supplementary File 1) which can be used by other researchers to study RPL using EHR data.

There are some limitations to our analysis. Although we filtered Control patients for no history of pregnancy loss, it is possible that some Control patients had previous pregnancy losses that were not recorded in EHR data. If so, some of our results could be underestimating the true effect size. Additionally, there were some observed associations with no clear clinical basis (e.g., poisoning, disturbances of sulphur-bearing amino-acid metabolism). Such associations may have arisen by chance owing to the large number of comparisons we made or due to confounding given that we could not control for numerous potential social and behavioral risk factors. We included data on the RPL and Control counts for each diagnosis in our results so readers can contextualize these findings by the numbers (Supplementary Files 2 & 3). Another limitation is that both UCSF and Stanford are academic medical centers in a similar region of California, so patients in these EHR databases are not representative of pregnant individuals in the general population. While our inter-center validation demonstrates some generalizability, further work is needed to assess generalizability in other populations^76,77^. Lastly, there may be diagnosis-specific differences in how often RPL vs Control patients were evaluated for each diagnosis. This is somewhat alleviated with the healthcare utilization sensitivity analysis, but to directly address this, further studies would need to consider the unique data provenance of each diagnosis^78^. This would require a targeted study of an individual association, instead of the large-scale agnostic approach that we applied here. The goal of our study was breadth, with the hope that we could generate hypotheses that would open up avenues for further research to investigate each one more deeply.

In total, our association analysis identified 48 diagnoses that were significantly positively associated with RPL at both UCSF and Stanford. This includes several menstrual abnormalities, spanning from absent/infrequent menstruation to excessive/frequent menstruation. Prior studies with smaller sample sizes found similar menstrual associations, but their results did not reach significance. Our study included 61,774 individuals, and our menstrual abnormality results were quite significant (all *p*-values<0.001 from their respective GAMs). We also saw a strong positive association with infertility – both the broad “infertility” diagnosis and more specific infertility-associated diagnoses including endometriosis, PCOS, and ovarian failure/dysfunction. This adds additional weight to the longstanding but contentious hypothesis that RPL and infertility could be connected. Future research can dive more deeply into plausible biological mechanisms for these associations. Lastly, subsequent studies should evaluate whether the documented associations will help predict patients at high risk of RPL and inform therapeutic strategies to help these patients.

## Methods

### Patient selection

Patients were selected from the UCSF Observational Medical Outcomes Partnership (OMOP) de-identified EHR database. UCSF OMOP contains data from 6,400,834 patients spanning 1982–2022. Patients were included in the RPL group if they had an RPL diagnosis or at least two pregnancy losses. Patients could fulfill the latter criterion by either (1) having a pregnancy loss after a recorded “history of pregnancy loss” or (2) having two pregnancy losses recorded at least 90 days apart from each other. The 90 day cutoff was chosen to ensure that the pregnancy loss records referred to two separate pregnancy losses, and not multiple records for the same loss. Patients were included in the Control group if they had a record of uncomplicated live-birth. Patients were excluded from the Control group if they had any record of: pregnancy loss, preterm birth, preterm labor, preterm rupture of membranes, multiple gestation with loss, molar pregnancy, or extrauterine pregnancy. All included RPL and Control patients were then filtered based on demographics, EHR data quality, and EHR data sufficiency, as summarized in Figure 2.

To identify which patients had records of RPL, live-birth, or adverse pregnancy outcomes, we curated lists of OMOP concepts for each pregnancy outcome. Curation was based on string-matching with search terms, and then manual review with guidance from clinical experts. Each OMOP concept list is included in Supplementary File 1. This can be used as a reference in other OMOP-based studies of RPL.

### Addressing sex and gender data limitations

Transgender, non-binary, and gender-diverse individuals are sometimes excluded from pregnancy research. This exclusion can lead to reduced generalizability of findings, erasure of patients’ identities, and barriers to high-quality clinical care^79^. Part of the problem is the lack of accurate gender information in EHR databases^80,81^. The standardized OMOP database structure contains a gender_id column which often is used for biological sex and has two standard options: female and male^82^.

In this study, we employed a couple of strategies to work around these limitations. For the demographic filtering step of the patient selection process, patients were included if (1) their gender_id value was female or (2) they had any record of incident pregnancy, regardless of their gender_id value. We also curated a list of OMOP concepts that may indicate a patient is transgender, non-binary, or gender-diverse (Supplementary File 1). After querying patients’ records for these concepts, we updated their gender information accordingly.

### Diagnosis querying and aggregation

Patients’ ICD-based diagnostic histories were queried. Any ICD9, ICD9-CM, ICD10, or ICD10-CM diagnosis occurring before RPL onset (or before first live-birth for Control patients) up until a year afterwards was included. All diagnoses were then aggregated using Phecodes^83^. Phecodes (https://phewascatalog.org/phecodes) provides a crosswalk for assembling ICD9 and ICD10 diagnoses into clinically-relevant phenotypes. There are two main advantages to this: (1) Analogous ICD9 and ICD10 diagnoses can be tested together, instead of arbitrarily separately. (2) The ICD hierarchy has many levels of specificity, and Phecodes aggregation allows us to group together similar diagnoses that are unnecessarily specific for our analysis.

### Visualizing patients’ EHR patterns with UMAP

To visualize overarching diagnosis patterns in patients, we applied UMAP to all non-pregnancy-related diagnoses. First, we created a dataframe where each row was a patient and each column was a diagnosis. All diagnoses occurring in at least 1 RPL or Control patient were included. For each patient, the presence or absence of each diagnosis in their records was one-hot-encoded. Next, we filtered out any diagnoses in the Phecodes category “pregnancy complications”. This was to ensure that the subsequent dimensionality reduction was based on diagnoses leading to the outcome (RPL or live-birth), instead of diagnoses indicating the outcome. We applied UMAP to the dataframe using the R package umap^84^. This reduced the dimensionality of the diagnosis data into two dimensions. We visualized the resulting UMAP coordinates using the R package ggplot2^85^, where points were colored based on whether the patient was in the RPL or Control group. We tested whether the UMAP coordinate distributions were significantly different between RPL and Control patients using Mann-Whitney U tests. The visualization coloring and coordinate distribution testing was also carried out for comparing patients <35 vs patients 35+. Additionally, we visualized UMAP coordinates across patients’ race, ethnicity, number of visits, years in EHR, and number of diagnoses (Supplementary Figures 2–5).

### Association analysis

For all diagnoses occurring in at least one RPL or Control patient, we implemented a case-control study to test the associations between each diagnosis and RPL. Crude associations were computed using logistic regression models. We used the glm() function in base R^86^. Confounder-adjusted associations were computed using GAMs. We used the gam() function in the R package mgcv^87^. The model covariates were maternal age, race, and ethnicity. A smoothing spline was applied to age to capture the non-linear relationship between age and pregnancy loss^37^. Race and ethnicity were included to mitigate potential confounding from social determinants of health that may be correlated with race/ethnicity, including the likelihood that patients receive diagnoses (both RPL and non-RPL diagnoses). All *p*-values were adjusted for multiple testing using the Benjamini-Hochberg method. Association results were visualized with a Manhattan plot and a Volcano plot, using the R package ggplot2^85^.

### Age-stratified analysis

Given the significant role that age plays in pregnancy loss^37^ and in health broadly, we hypothesized that RPL association patterns may vary across age strata. We compared RPL associations in younger vs older patients using an age-stratified analysis. To do this, we separated patients into two strata based on whether they were <35 or 35+ at the first record of RPL onset (or first live-birth for Control patients). Then, we ran two separate association analyses for <35 and 35+ patients. Results were visualized with a Log-Log plot using the R package ggplot2^85^.

### Healthcare utilization sensitivity analysis

We hypothesized that healthcare utilization could confound our association results. To investigate this, we re-ran our association analysis with the number of visits (in the study window) included as a covariate, in addition to the previously included covariates. Results were visualized with a Log-Log plot using the R package ggplot2^85^.

### External validation using the Stanford EHR database

We queried the Stanford OMOP de-identified EHR database containing 3,604,034 patients spanning 1999–2022^88^. Patients were selected using the same criteria as described for the UCSF patients. The number of Stanford patients remaining at each step of the selection process is summarized in Supplementary Figure 1. All three association analyses (main, age-stratified, healthcare utilization) were repeated on the Stanford data. Stanford results were compared to UCSF results by (1) quantifying overlaps between significant positive and negative associations, (2) computing correlations between odds ratios, and (3) visualizing results with Log-Log plots using the R package ggplot2^85^.

### Ethical approval

This study was approved by the Institutional Review Board of University of California San Francisco (#17–22929) and by the Institutional Review Board of Stanford University (#39225).

## Data Availability

All EHR concept lists are in Supplementary File 1. UCSF and Stanford diagnosis association results are in Supplementary Files 2 and 3, respectively. In our association analyses, some diagnoses had very low (<10) patient counts. To maintain patient de-identification, exact counts, odds ratios, and *p*-values are redacted for those diagnoses in Supplementary Files 2 and 3. UCSF-affiliated individuals can request access to UCSF EHR data by contacting UCSF Information Commons (Info.Commons@ucsf.edu). Stanford’s EHR data is managed through the Stanford Research Repository (https://med.stanford.edu/starr-tools.html).
